# Associations of perceived changes in work due to digitalization and the amount of digital work with job strain among physicians: a national representative sample

**DOI:** 10.1186/s12911-023-02351-9

**Published:** 2023-11-08

**Authors:** Lotta Virtanen, Anu-Marja Kaihlanen, Petra Saukkonen, Jarmo Reponen, Tinja Lääveri, Tuulikki Vehko, Peppiina Saastamoinen, Johanna Viitanen, Tarja Heponiemi

**Affiliations:** 1 Department of Public Health and Welfare, Finnish Institute for Health and Welfare, P.O.Box 30, 00271 Helsinki, Finland; 2 Research Unit of Health Sciences and Technology, University of Oulu, P.O.Box 5000, 90014 Oulu, Finland; 3 Medical Research Center, Oulu University Hospital and University of Oulu, P.O.Box 8000, 90014 Oulu, Finland; 4 Department of Infectious Diseases, University of Helsinki and Helsinki University Hospital, P.O.Box 700, 00029 Helsinki, Finland; 5 Department of Computer Science, Aalto University, P.O.Box 15400, 00076 Espoo, Finland; 6 Finnish Medical Association, P.O.Box 49, 00501 Helsinki, Finland

**Keywords:** Occupational stress, Physicians, Health information systems, Electronic health records, Telemedicine

## Abstract

**Background:**

Physicians’ work is often stressful. The digitalization of healthcare aims to streamline work, but not all physicians have experienced its realization. We examined associations of perceived changes in work due to digitalization and the amount of digital work with job strain among physicians. The moderating role of the length of work experience was investigated for these associations.

**Methods:**

We used representative survey data on Finnish physicians’ (*N* = 4271) experiences of digitalization from 2021. The independent variables included perceptions on statements about work transformations aligned with digitalization goals, and the extent that information systems and teleconsultations were utilized. Stress related to information systems (SRIS), time pressure, and psychological stress were the dependent variables. We analyzed the associations using multivariable linear and logistic regressions.

**Results:**

Respondents had a mean SRIS score of 3.5 and a mean time pressure score of 3.7 on a scale of 1–5. Psychological stress was experienced by 60%. Perceptions associated with higher SRIS comprised disagreements with statements asserting that digitalization accelerates clinical encounters (*b* = .23 [95% CI: .16–.30]), facilitates access to patient information (*b* = .15 [.07–.23]), and supports decision-making (*b* = .11 [.05–.18]). Disagreement with accelerated clinical encounters (*b* = .12 [.04–.20]), and agreements with patients’ more active role in care (*b* = .11 [.04–.19]) and interprofessional collaboration (*b* = .10 [.02–.18]) were opinions associated with greater time pressure. Disagreeing with supported decision-making (OR = 1.26 [1.06–1.48]) and agreeing with patients’ active role (OR = 1.19 [1.02–1.40]) were associated with greater psychological stress. However, perceiving improvements in the pace of clinical encounters and access to patient information appeared to alleviate job strain. Additionally, extensive digital work was consistently linked to higher strain. Those respondents who held teleconsultations frequently and had less than 6 years of work experience reported the greatest levels of time pressure.

**Conclusions:**

Physicians seem to be strained by frequent teleconsultations and work that does not meet the goals of digitalization. Improving physicians’ satisfaction with digitalization through training specific to the stage of career and system development can be crucial for their well-being. Schedules for digital tasks should be planned and allocated to prevent strain related to achieving the digitalization goals.

**Supplementary Information:**

The online version contains supplementary material available at 10.1186/s12911-023-02351-9.

## Background

Physicians are often exposed to job strain, such as time pressure and psychological stress [1–3], which can be described as unpleasant and potentially harmful psychological reactions to work [4]. Job strain can impair the cognitive functions that are essential for physicians’ work [5], and has been associated with a decrease in the quality and safety of care [6, 7]. Additionally, prolonged job strain can partly explain the high turnover and shortage of physicians [8, 9]. The possible severe consequences of job strain on physicians’ health should also not be underestimated [8].

Exposure to job strain arises from the significant job demands inherent in physicians’ work, such as managing a heavy workload, staying updated with expanding medical knowledge, and adapting to atypical work arrangements [3, 5, 10]. Additionally, the profession is characterized by greater emotional demands when confronting distressing situations, challenging patients, and the need to conceal one’s emotions [10]. Nevertheless, these demands may not always translate into job strain. The Job Demands-Control Model [11] identifies two distinct high-demand work profiles: high strain and healthy active. High strain job involves excessive demands that often surpass the individual’s ability to exert control over work-related decisions, potentially leading to elevated job strain. In contrast, healthy active job represents ideal working conditions where high demands remain manageable due to a greater degree of control [11]. The Job Demands-Resources Model [12] expands this perspective by recognizing that, overall, adequate resources provided in the workplace could mitigate the adverse effects of demanding work on employees’ well-being.

The digitalization of healthcare may have the potential to serve as a job resource by streamlining work [13–17]. Digital work utilizes digital health technologies (DHTs): health information systems (HISs) such as electronic health records (EHRs), clinical decision support systems (CDSSs), and telemedicine technology with video, chat, or phone connection for teleconsultations with patient and remote collaboration between professionals [18]. Additionally, wearable devices and digital services can be provided for patient self-care, and patients and professionals can exchange information through patient portals [18]. The strategic goals of implementing these DHTs in healthcare include activating patients’ role in care, improving access to patient data, supporting clinical decision-making, making clinical encounters more efficient, progressing with interprofessional collaboration, and enhancing the possibilities for preventive care [16, 19].

Digitalization, however, represents a significant process of change in the nature of work and professional culture [17, 20, 21]. This transformation means that working days may increasingly blend computer-based tasks with cognitive tasks [22, 23]. Professional performance can now be artificial intelligence (AI) -assisted, with prompts, optimization, and alerts integrated into HISs [24]. Furthermore, as patients gain greater access to health information through the internet and self-generated health data, the physician–patient relationship may become less hierarchical [17, 20]. Thus, physicians’ guiding role and collaboration with patients can increase [17, 20].

A theoretical framework by Day et al. [25] applies job strain models to technology-driven organizations, when suggesting that the way in which an employee perceives digitalization altering work—such as in terms of workload, access to information, work control, communication, and collaboration—could determine whether digitalization acts as a stressor or a resource for well-being at work. Based on the framework, we can hypothesize that physicians might experience increased strain if they perceive negative changes in their work due to digitalization. Conversely, physicians might experience reduced strain if they perceive these changes as improvements to work and alignment with the goals of digitalization.

Previous research has suggested that many physicians may not perceive that the alterations correspond with the goals of digitalization [26]. Although the connection between these perceptions and job strain is not yet fully known, studies imply that changes by DHTs could act as additional stressors in physicians’ work [27–41]. Updates to or new implementations of HISs along with difficult, malfunctioning equipment and software have created a new type of job strain known as stress related to information systems (SRIS) [28–31]. Poor usability of EHRs, stemming from shortcomings in their design and functionality that complicate effective use, has been associated with greater time pressure and psychological stress [30, 32–34]. Moreover, digital work seems to exacerbate work disruptions due to the time spent documenting and solving technical issues in EHRs [35–37] and reviewing irrelevant pop-ups in CDSSs [38]. Compared to in-person encounters, teleconsultations may require more clinical activity from physicians and efforts to build a relationship with the patient from a distance [39–41], which can be stressful [42, 43].

Furthermore, the amount of digital work involved in the job might contribute to job strain. The use of several different HISs can increase the complexity of the work and has been associated with job strain [30, 33, 44–46]. Similarly, employees who engage with technology more frequently may confront a more intense work pace, interruptions, and greater cognitive load, potentially exposing them to increased job strain compared to those who have lower levels of technology engagement [13, 47, 48].

It is essential to gain a more precise understanding of the effects of healthcare digitalization on job strain, particularly when the attractiveness and retention of healthcare professionals are under scrutiny [9, 49]. Although the digitalization of healthcare work has become a global phenomenon [50], it may be meaningful to investigate its effects in a country that has a history of being at the forefront of digitalization. This research evidence could guide the planning and development of digital work practices and promote a healthy work environment in healthcare organizations. Additionally, the findings could offer valuable insights for countries in earlier stages of digitalization.

Finland is known for its long-term provision of national digital health services and extensive use of DHTs [51–53]. The intensity of use of most HISs in Finnish healthcare organizations is exceptionally high, and teleconsultations were performed before the COVID-19 pandemic [52]. Despite nationwide practices, not all systems seamlessly integrate with each other, which can increase duplicated documentation work and hamper continuity of care [54]. For example, many areas share a common core EHR system, but there are also ancillary systems, such as in emergency departments, diagnostics, and operative units that are partly incompatible between sectors, organizations, and units [52, 54, 55].

The shortage of physicians also affects Finland, especially in the public sector and sparsely populated areas [56]. Because of the universal access to care in the country, the public sector (i.e., municipal health centers and public hospitals) is the most important employer of physicians and is also responsible for the most demanding treatment [57, 58]. Many physicians work in both the public and private sectors. The main place of employment for 16% of physicians is the private sector, such as private practices or occupational healthcare, and 11% of physicians work in other areas such as state-supported student healthcare [57, 58].

This study aimed to examine the associations of perceived changes in work due to digitalization and the amount of digital work with job strain among Finnish physicians. Different aspects of job strain—SRIS, time pressure, and psychological stress—were considered, in order to obtain a comprehensive overview of psychological pressures and their related factors. We also investigated whether the length of work experience moderated possible associations, as it may shape experiences of strain caused by transforming work [35, 37–42]. We addressed the following research questions:

1. Do the perceived changes in work due to digitalization and the amount of digital work potentially predict a) SRIS, b) time pressure, and c) psychological stress?
2. Does the length of work experience moderate these potential associations?

## Methods

### Study design

We performed a cross-sectional study of the data collected for the *Electronic Health Record Systems as a Tool for Physicians 2021 Study* [59]. The primary aim of the data collection was to monitor physicians’ experiences concerning the usability of HISs in a monitoring project in Finland [60].

### Data collection

**Fig. 1:**
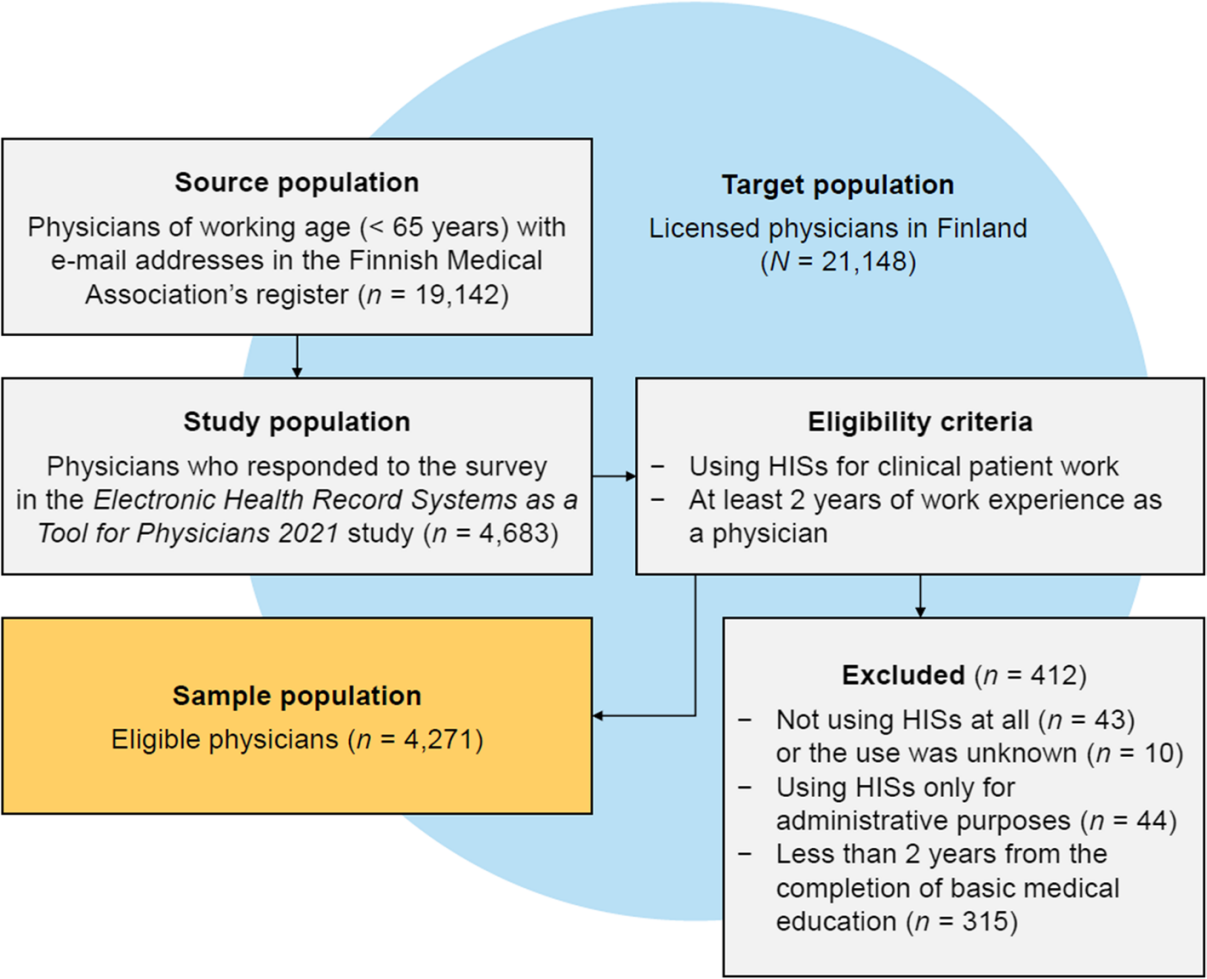
Formation of the sample population. ﻿The number of physicians in the target and source population was obtained from the Finnish Medical Association [57]

An online survey [61] was conducted between January and March 2021. The development and validation of the survey is described elsewhere [62, 63]. Physicians of working age were identified from the Finnish Medical Association’s register and invited to participate by e-mail (Fig. 1). Up to three reminders were sent, and the response rate was 25%. The sample was narrowed to those who used HISs in clinical patient work and had at least 2 years of work experience to be able to assess longer-term changes in work due to digitalization.

The Finnish National Board on Research Integrity [64] has outlined that the administration of surveys to gather respondents’ opinions, which are not anticipated to result in harm, does not necessitate a statement from the ethics committee, as in the case of this study. All respondents received written information about the study, participated voluntarily, and provided informed consent by clicking the consent box on the first page of the survey.

### Dependent variables

*SRIS* was measured from a mean of two items asking how often during the past 6 months the respondent had been distracted by, worried about, or burdened by 1) changing HISs, and 2) awkward, poorly functioning IT equipment or software (Cronbach’s alpha [α] = 0.76). The responses were rated using a Likert response format: 1 = very rarely or never, 2 = quite rarely, 3 = occasionally, 4 = quite often, 5 = very often or constantly. This instrument has been previously used in studies among physicians [28–30].

*Time pressure* was assessed from the mean of two items asking how often during the past 6 months the respondent had been distracted by, worried about, or burdened by 1) constant rush and pressure due to uncompleted work, and 2) not enough time to perform work properly (α = 0.91). The response options were rated with the same alternatives as for the SRIS. The instrument was derived from the Harris stress index [65], and has been validated [66] and used in studies among physicians [28, 33, 45].

*Psychological stress* was based on one question about whether the respondent was experiencing stress at that moment, described as feeling tense, restless, nervous, or anxious, or finding it hard to sleep because of constant worry about things. The response options were: 1 = not at all, 2 = just a little, 3 = to some extent, 4 = quite a lot, and 5 = very much, and were recorded as 0 = no (response options 1–2) and 1 = yes (3–5), in a similar way to a previous study of physicians [45]. This question has been developed from a symptom checklist of mental health screening and clinical experiences of occupational healthcare, and has been validated and widely used [67].

### Independent variables

Perceived changes in work due to digitalization were measured by respondents’ opinions on the following statements on how the digitalization of healthcare had changed their work in the past 3 years:

1. Patients have assumed a more active role in their treatment (*more active role of patients*).
2. It has become easier to obtain information on patients (*facilitated access to patient information*).
3. Intelligent CDSSs support a physician’s work (*supported decision-making*).
4. Consultations with patients have become faster (*accelerated clinical encounters*).
5. Interprofessional collaboration has progressed (*progressed interprofessional collaboration*).
6. Possibilities for preventive work have improved (*improved possibilities for preventive work*).

The statements were rated: 1 = fully agree, 2 = somewhat agree, 3 = neither agree nor disagree, 4 = somewhat disagree, 5 = fully disagree, which were recoded as 1 = neutral (response option 3), 2 = agree (1–2), and 3 = disagree (4–5). The statements were developed by expert researchers based on the Finnish strategic goals of healthcare digitalization [19] and were piloted. These statements have been successfully used previously and found to interplay with work-related factors [26].

The amount of digital work was assessed by two variables. For *the number of HISs in daily use,* the respondents were asked how many different clinical systems they logged into daily in their clinical work. The variable was recoded as 1 = two systems or fewer, and 2 = three or more. *The frequency of teleconsultations* was based on the respondents’ assessment of how much their main employment involved teleconsultation with patients. The response options were 1 = not at all, 2 = a little, 3 = to some extent, 4 = much, 5 = very much, and recoded as 1 = not at all–to some extent and 2 = much–very much.

### Background variables

The* length of work experience* was based on the year of completion of the basic medical education, encoded as 1 = 2–5 years, 2 = 6–9 years, 3 = 10–19 years, and 4 = 20 years or longer. The employment rate of physicians is high in Finland [68], so the time between the year of graduation and the survey response could be expected to describe the length of employment quite well.

Other background variables (relevant demographic and professional factors) and the original questions of the study variables are described in Additional file 1.

### Data analysis

We employed multiple imputations (*n* = 5 datasets) using an automatic method [69] for all study variables to generate valid statistical inferences for missing data [70]. Details of the missing data and the multiple imputations are presented in Additional file 2. All the analyzed and displayed data was derived from the means of the estimates in the imputed datasets to obtain a pooled estimate [71]. We described the sample and prevalence of job strain with descriptive statistics.

We performed separate linear regression analysis to examine the associations of independent variables (i.e., perceived changes in work due to digitalization and the amount of digital work) with SRIS and time pressure. Additionally, we employed logistic regression analysis to examine the odds of psychological stress based on the same independent variables. In univariable models, we measured the crude associations of every single independent variable with a) SRIS, b) time pressure, and c) psychological stress. We then used the enter method to assess whether these associations would change when all the independent variables were simultaneously added to the multivariable models. This approach allowed us to evaluate the predictive power of the models, address real-world complexities, and derive more accurate values for the variables of interest compared to a model including only statistically significant variables [72]. We also identified potential confounding variables among the background factors, guided by previous research that has linked factors related to individuals [1, 4, 73], the experience and quality of EHRs [30, 33, 74], and the work environment [4, 29, 31, 45] to job strain. We determined which of these background factors might act as confounders in our data, using a cut-off of 10% for the change-in-estimate criterion [75]. This meant that we assessed whether the inclusion versus exclusion of these factors in the models resulted in an increase or decrease of 10% or more in the estimated coefficients of the independent variables. Thus, we adjusted the multivariable SRIS and time pressure models for all background variables and the multivariable psychological stress model for gender, experience with the current EHR in use, the EHR grade, and the working sector. The assumptions for linear and logistic regression, including the absence of multicollinearity [76], were met. Furthermore, we tested the potential interaction terms by separately adding interactions between the length of work experience and each independent variable to the multivariable models.

The analysis was carried out using IBM SPSS Statistics software version 28 with a significance level of α < 0.05.

## Results

The sample comprised 4271 physicians (Table 1), of whom 51% had the longest work experience of at least 20 years and 12% the shortest work experience of 2–5 years. Although the majority (62%) reported inpatient care as their primary working unit, many physicians worked in diverse environments. The mean SRIS (*M* = 3.5, *SD* = 1.1) was slightly lower compared to the mean time pressure (*M* = 3.7, *SD* = 1.1). Psychological stress was experienced by 60% of respondents. Among the statements regarding the changes in work due to digitalization, the physicians disagreed the most about accelerated clinical encounters (67%), whereas they agreed most with statements about the more active role of patients (47%) and facilitated access to patient information (44%). Almost two-fifths (37%) used at least three HISs daily, and 16% reported that they conducted many teleconsultations.

**Table 1:** Characteristics of the studied physicians (*N* = 4271)

Characteristics	Value
*Background*	
Age, n (%)	
< 35	681 (15.9)
35–44	1190 (27.9)
45–54	1131 (26.5)
55–64	1269 (29.7)
Gender, n (%)	
Male	1459 (34.1)
Female	2771 (64.9)
Other	41 (1.0)
Length of work experience, n (%)	
2–5 years	526 (12.3)
6–9 years	473 (11.1)
10–19 years	1102 (25.8)
20 years or longer	2170 (50.8)
Experience with the current EHR, n (%)	
< 1 year	1184 (27.7)
1–3 years	1239 (29.0)
> 3 years	1848 (43.3)
EHR grade, n (%)	
Low	2743 (64.2)
High	1512 (35.4)
No opinion	16 (0.4)
Working sector, n (%)	
Public hospital	2632 (61.6)
Public health center	830 (19.4)
Private clinic or hospital	599 (14.0)
Other	210 (4.9)
Working unit, n (%)	
Inpatient	2661 (62.3)
Outpatient	539 (12.6)
Emergency department	161 (3.8)
Operative, intensive care, or delivery room	601 (14.1)
Diagnostics	146 (3.4)
Administrative	163 (3.8)
*Job strain*	
SRIS^a^, mean (SD)	3.5 (1.1)
Time pressure^a^, mean (SD)	3.7 (1.1)
Psychological stress, n (%)	
No	1700 (39.8)
Yes	2571 (60.2)
*Perceived changes in work due to digitalization*	
More active role of patients, n (%)	
Neutral	1286 (30.1)
Agree	1984 (46.5)
Disagree	1001 (23.4)
Facilitated access to patient information, n (%)	
Neutral	946 (22.2)
Agree	1880 (44.0)
Disagree	1445 (33.8)
Supported decision-making, n (%)	
Neutral	1851 (43.4)
Agree	894 (20.9)
Disagree	1526 (35.7)
Accelerated clinical encounters, n (%)	
Neutral	998 (23.3)
Agree	422 (9.9)
Disagree	2851 (66.8)
Progressed interprofessional collaboration, n (%)	
Neutral	1447 (33.9)
Agree	1473 (34.5)
Disagree	1351 (31.6)
Improved possibilities for preventive work, n (%)	
Neutral	1937 (45.4)
Agree	808 (18.9)
Disagree	1526 (35.7)
*Amount of digital work*	
Number of HISs in daily use, n (%)	
≤ 2	2691 (63.0)
≥ 3	1580 (37.0)
Frequency of teleconsultations, n (%)	
Not at all–to some extent	3602 (84.3)
Much–very much	669 (15.7)

*EHR* Electronic health record, *SRIS* Stress related to information systems

^a^Scale ranged between 1 and 5, where a higher score indicated greater job strain. Variables were based on the questionnaire of the *Electronic Health Record Systems as a Tool for Physicians 2021 Study* [61]

### Variables associated with SRIS

Table 2 shows the results of the linear regressions for SRIS. In the multivariable model, when controlling for the other variables, physicians who disagreed with the statement about accelerated clinical encounters had on average 0.23 (95% CI [0.16, 0.30]) points higher SRIS compared to those who perceived the statement as neutral. Similarly, disagreements with the statements about facilitated access to patient information (*b* = 0.15, 95% CI [0.07, 0.23]) and supported decision-making (*b* = 0.11, 95% CI [0.05, 0.18]) were associated with higher SRIS. A large amount of digital work was also significantly associated with higher SRIS. In turn, physicians who agreed with the statements about facilitated access to patient information (*b* =  − 0.09, 95% CI [− 0.16, − 0.02]) and improved interprofessional collaboration (*b* =  − 0.07, 95% CI [− 0.14, − 0.01]) had slightly lower SRIS on average compared to those who perceived the statements as neutral.

**Table 2:** Results of the univariable and multivariable linear regression analyses for SRIS

Variable	Univariable model			Multivariable model		
	*b*	95% CI	*P* value	*b*	95% CI	*P* value
*Perceived changes in work due to digitalization*						
More active role of patients						
Neutral	ref			ref		
Disagree	.13	.04, .22	.003	− .05	− .14, .03	.20
Agree	− .10	− .18, − .03	.01	.01	-.05, .07	.76
Improved possibilities for preventive work						
Neutral	ref			ref		
Disagree	.24	.17, .31	< .001	.01	− .06, .08	.73
Agree	− .24	− .33, − .15	< . 001	.01	− .07, .09	.77
Progressed interprofessional collaboration						
Neutral	ref			ref		
Disagree	.31	.23, .39	< .001	− .03	− .11, .04	.43
Agree	− .26	− .33, − .18	< .001	− .07	− .14, − .01	.03
Supported decision-making						
Neutral	ref			ref		
Disagree	.38	.31, .45	< .001	.11	.05, .18	< .001
Agree	− .20	− .28, − .11	< .001	− .03	− .11, .04	.37
Facilitated access to patient information						
Neutral	ref			ref		
Disagree	.57	.49, .65	< .001	.15	.07, .23	< .001
Agree	− .31	− .39, − .23	< .001	− .09	− .16, − .02	.008
Accelerated clinical encounters						
Neutral	ref			ref		
Disagree	.63	.56, .71	< .001	.23	.16, .30	< .001
Agree	− .25	− .37, − .13	< .001	− .03	− .13, .07	.58
*Amount of digital work*						
Number of HISs in daily use						
≤ 2	ref			ref		
≥ 3	.52	.46, .59	< .001	.24	.18, .29	< .001
Frequency of teleconsultations						
Not at all–to some extent	ref			ref		
Much–very much	.06	− .03, .15	.21	.15	.07, .22	< .001

*N* = 4271. *SRIS* Stress related to information systems, *b* unstandardized beta coefficient, which indicated how many points on average the SRIS score (scale 1–5) increased or decreased in a certain group compared to a reference group; *CI* Confidence interval, *ref*. Reference, *HISs* Health information systems. The univariable model measured the independent effect of each variable with SRIS separately. The multivariable model measured the joint effects of the variables of perceived changes in work due to digitalization and amount of digital work with SRIS, adjusted by gender, length of work experience, experience with the current EHR, EHR grade, location of employment, working sector, and working unit. The multivariable model was significant, *F* (35, *N* = 4271) = 83.79, *P* < .001, explaining 41% (R^2^) of the variance in SRIS. Variables were based on the questionnaire of the *Electronic Health Record Systems as a Tool for Physicians 2021 Study* [61]

### Variables associated with time pressure

Table 3 shows the results of the linear regressions for time pressure. In the multivariable model, when controlling for the other variables, physicians who disagreed with the statement about accelerated clinical encounters had on average 0.12 (95% CI [0.04, 0.20]) points greater time pressure compared to those who perceived the statement as neutral. Physicians who agreed that patients had taken a more active role (*b* = 0.11, 95% CI [0.04, 0.19]) and that interprofessional collaboration had progressed (*b* = 0.10, 95% CI [0.02, 0.18]) had greater time pressure compared to those with a neutral perception of the statements. A large amount of digital work was also significantly associated with greater time pressure.

**Table 3:** Results of the univariable and multivariable linear regression analyses for time pressure

Variable	Univariable model			Multivariable model		
	*b*	95% CI	*P* value	*b*	95% CI	*P* value
*Perceived changes in work due to digitalization*						
More active role of patients						
Neutral	ref			ref		
Disagree	.12	.03, .21	.007	.01	− .09, .10	.92
Agree	.14	.07, .22	< .001	.11	.04, .19	.004
Improved possibilities for preventive work						
Neutral	ref			ref		
Disagree	.18	.11, .26	< .001	.08	− .004, .17	.06
Agree	.04	− .05, .13	.38	.06	− .03, .15	.21
Progressed interprofessional collaboration						
Neutral	ref			ref		
Disagree	.17	.09, .25	< .001	.02	− .07, .11	.66
Agree	.11	.04, .19	.004	.10	.02, .18	.01
Supported decision-making						
Neutral	ref			ref		
Disagree	.12	.05, .19	.001	.05	− .03, .12	.23
Agree	.11	.03, .20	.01	.04	− .05, .12	.41
Facilitated access to patient information						
Neutral	ref			ref		
Disagree	.15	.06, .24	< .001	.004	− .09, .10	.92
Agree	− .10	− .19, − .02	.02	− .06	− .14, .02	.15
Accelerated clinical encounters						
Neutral	ref			ref		
Disagree	.28	.20, .35	< .001	.12	.04, .20	.003
Agree	− .14	− .26, − .02	.03	− .09	− .21, .02	.12
*Amount of digital work*						
Number of HISs in daily use						
≤ 2	ref			ref		
≥ 3	.29	.22, .35	< .001	.17	.11, .23	< .001
Frequency of teleconsultations						
Not at all–to some extent	ref			ref		
Much–very much	.19	.10, .28	< .001	.22	.14, .31	< .001

*N* = 4271, *b* Unstandardized beta coefficient, which indicated how many points on average the time pressure score (scale 1–5) increased or decreased in a certain group compared to a reference group; *CI* Confidence interval, *ref.* Reference, *HISs* Health information systems. The univariable model measured the independent effect of each variable with time pressure separately. The multivariable model included simultaneously the variables of perceived changes in work due to digitalization and amount of digital work with time pressure, adjusted by gender, length of work experience, experience with the current EHR, EHR grade, location of employment, working sector, and working unit. The multivariable model was significant, *F *(35, *N* = 4271) = 20.84, *P* < .001, explaining 15% (R^2^) of the variance in time pressure. Variables were based on the questionnaire of the *Electronic Health Record Systems as a Tool for Physicians 2021 Study* [61]

### Variables associated with psychological stress

Table 4 presents the results of the logistic regressions for psychological stress. In the multivariable model, when controlling for the other variables, physicians who disagreed with the statement about supported decision-making and agreed with the statement about the more active role of patients had on average 1.26 (95% CI [1.06, 1.48]) and 1.19 (95% CI [1.02, 1.40]) times greater odds of psychological stress, respectively, compared to those with a neutral perception. However, physicians who agreed with facilitated access to patient information (OR = 0.84, 95% CI [0.70, 0.98]) and accelerated clinical encounters (OR = 0.72, 95% CI [0.56, 0.92]) had lower odds of psychological stress compared to their counterparts. The odds of psychological stress were 1.47 (95% CI [1.22, 1.76]) and 1.19 (95% CI [1.04, 1.47]) times greater for physicians who conducted many teleconsultations and used three or more HISs, respectively, compared to their counterparts.

**Table 4:** Results of the univariable and multivariable logistic regression analyses for psychological stress

Variable	Univariable model			Multivariable model		
	OR	95% CI	*P* value	OR	95% CI	*P* value
*Perceived changes in work due to digitalization*						
More active role of patients						
Neutral	ref			ref		
Disagree	1.15	.97, 1.37	.10	.99	.81, 1.20	.88
Agree	1.12	.97, 1.30	.12	1.19	1.02, 1.40	.03
Improved possibilities for preventive work						
Neutral	ref			ref		
Disagree	1.26	1.09, 1.45	< .001	1.15	.96, 1.38	.13
Agree	.87	.73, 1.02	.09	.92	.76, 1.11	.41
Progressed interprofessional collaboration						
Neutral	ref			ref		
Disagree	1.20	1.03, 1.40	.02	.97	.80, 1.17	.74
Agree	1.01	.87, 1.17	.96	1.11	.94, 1.31	.22
Supported decision-making						
Neutral	ref			ref		
Disagree	1.31	1.14, 1.32	< .001	1.26	1.06, 1.48	.007
Agree	.99	.84, .89	.91	1.02	.85, 1.23	.83
Facilitated access to patient information						
Neutral	ref			ref		
Disagree	1.11	.93, 1.32	.24	.87	.72, 1.06	.17
Agree	.76	.65, .89	< .001	.84	.70, .98	.04
Accelerated clinical encounters						
Neutral	ref			ref		
Disagree	1.30	1.12, 1.51	< .001	1.05	.89, 1.24	.58
Agree	.65	.52, .82	< .001	.72	.56, .92	.009
*Amount of digital work*						
Number of HISs in daily use						
≤ 2	ref			ref		
≥ 3	1.39	1.22, 1.58	< .001	1.19	1.04, 1.47	.01
Frequency of teleconsultations						
Not at all–to some extent	ref			ref		
Much–very much	1.45	1.36, 1.55	< .001	1.47	1.22, 1.76	< .001

*N* = 4271, *OR* The Odds Ratio indicated, on average, how many times greater or lower odds of psychological stress there were for a certain group compared to a reference group; *CI*   Confidence interval, *ref.* Reference, *HISs* Health information systems. The univariable model measured the independent effect of each variable with psychological stress separately. The multivariable model included simultaneously the variables of perceived changes in work due to digitalization and amount of digital work, adjusted by gender, experience with the current EHR, EHR grade, and working sector. The multivariable model was significant, χ^2^ (23, *N* = 4271) = 243.74, *P* < .001, explaining 8% (Nagelkerke R^2^) of the variance in psychological stress. The goodness-of-fit of the model was good (Hosmer–Lemeshow test with *P* > .05), and the model correctly classified 63% of cases. Variables were based on the questionnaire of the *Electronic Health Record Systems as a Tool for Physicians 2021 Study* [61]

### The moderating effect of the length of work experience

**Fig. 2:**
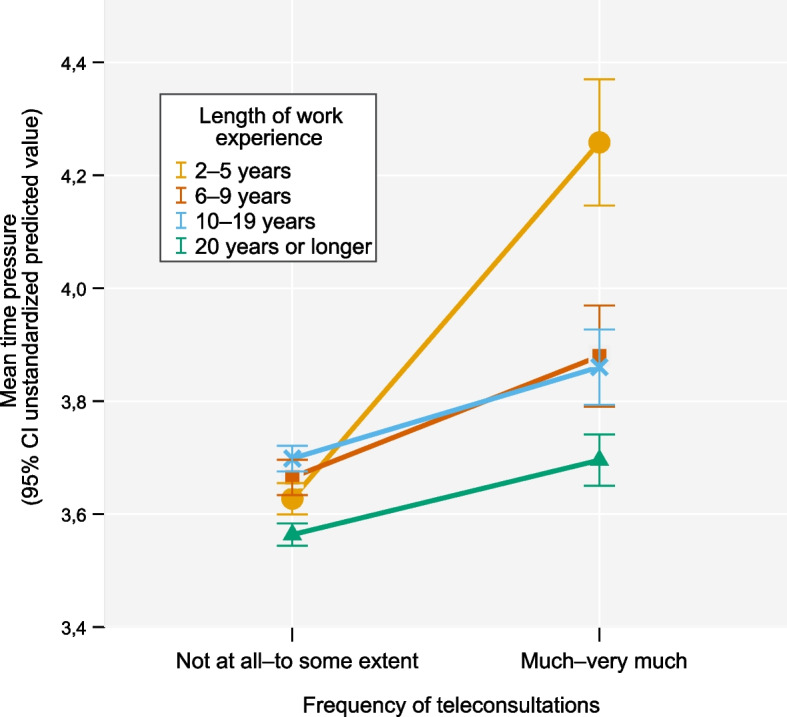
The moderating effect of the length of work experience on the time pressure model. Interaction between frequency of teleconsultations and the length of work experience for mean time pressure score with 95% CI among physicians (*N* = 4271)

We found a significant interaction effect between the frequency of teleconsultations and the length of work experience in terms of time pressure (Fig. 2). Among the physicians who conducted many teleconsultations, those who had worked as a physician for 2–5 years had on average greater time pressure compared to those who had worked 6–9 years (*b* =  − 0.43, 95% CI [− 0.80, − 0.07]), 10–19 years (*b* =  − 0.40, 95% CI [− 0.71, − 0.09]), or 20 years or longer (*b* =  − 0.46, 95% CI [− 0.75, − 0.17]), when controlling for the other variables.

## Discussion

In this study, we examined the associations of perceived changes in work due to digitalization and the amount of digital work with job strain among Finnish physicians, and the moderating role of the length of work experience. The physicians reported a relatively high degree of job strain, as they, on average, indicated experiencing SRIS and time pressure quite often, similarly to earlier Finnish studies [28–30]. Furthermore, 60% of physicians in our study experienced psychological stress at least to some extent, which is 14 percentage points higher than in the data collected 4 years earlier [45]. During this time frame, digitalization further accelerated [52, 53]. Our results suggest that all the perceived changes in work due to digitalization in recent years that we studied, except for the change related to preventive work, and the amount of digital work may predict job strain. Exposure to digital job strain may occur in the early stages of a physician’s career in particular.

We found higher SRIS among physicians who considered that digitalization had not accelerated clinical encounters, facilitated access to patient information, or supported decision-making. Greater time pressure was experienced by those who felt that clinical encounters were not accelerated, but who felt that patients were more active and interprofessional collaboration had been progressed. Those who considered that decision-making had not been supported by digitalization and that patients were activated seemed to have a greater chance of psychological stress. However, the experience of facilitated access to patient information was associated with lower SRIS and a lower chance of psychological stress; and experiences of improved interprofessional collaboration and accelerated clinical encounters were associated with lower SRIS or a lower chance of psychological stress, respectively. Moreover, the use of three or more HISs and frequent teleconsultations were consistently related to higher job strain. The length of work experience moderated the association between the frequency of teleconsultations and time pressure: physicians who frequently performed teleconsultations and had less than 6 years of work experience reported the greatest time pressure.

Our study involved a diverse group of physicians, including those who were dissatisfied with the changes in access to patient information and the pace of clinical encounters resulting from digitalization, as well as those who were content with these changes. It is noteworthy that these varying attitudes may strongly predict the level of SRIS, time pressure, or psychological stress. Since improved access to patient data is a fundamental aspect of HISs, our finding that its disagreement serves as a stressor may imply that some physicians perceive the use of HISs as too complex or that they may lack sufficient training, complementing previous studies [28–30, 77]. Additionally, those who are dissatisfied with the HISs may feel they disrupt clinical encounters, negating their time-saving benefits [35–37]. Our results are also in line with the broader literature on technostress, which suggests that digital technologies can introduce new demands at work due to their complexity and unreliability, the need for continuous learning, and related perceived unrealistic expectations of work efficiency [78, 79].

Nevertheless, our study also supports the framework by Day et al. [25], as it highlights the dual nature of physicians’ experiences with technology in relation to job strain. In our study, for some physicians, changes in access to patient information and clinical encounters resulting from digitalization appeared to serve as job resources, which may alleviate job strain. Digitalization-induced work resources, such as EHRs that support work and teleconsultations that increase job control and balance job demands, have also been identified as factors that may reduce job strain in previous Nordic studies [14, 30]. Since only a minority of the participants in our study appeared to be satisfied with digitalization, there is a critical need to enhance physicians’ positive perceptions through training [77] and developing DHTs that cater to the demands of routine work in Finland.

In our study, disagreement that CDSSs had supported decision-making was associated with higher SRIS and, on average, a 26% greater chance of psychological stress compared to those who were neutral about the change. Possible explanations for this might be the lack of trust in the accuracy of data collected by AI and that frequent verification is required [44], or the disruptive presence of irrelevant pop-ups in CDSSs [38]. The concept of alert fatigue has been used to describe a situation where the ability to respond to an alert is declining due to repeated exposure to inconsequential alerts [80]. Indeed, lacking or poorly constructed reminders and CDSSs that are not properly integrated in important fields of medicine may become counterproductive if physicians are unable to cognitively discern and absorb essential reminders from irrelevant ones.

Our study suggests that some of the benefit-seeking changes in work related to digitalization could increase physicians’ job strain. For example, we found that patients taking an active role was associated with greater time pressure and psychological stress for the physicians. Patient self-care has previously been linked to lower use of healthcare resources [81, 82], but our results suggest that digital self-care could increase physicians’ workloads. Supporting our results, previous studies have found that work efficiency is compromised because active patients seek assistance for the same issue—not only through digital contact but also through other channels [21, 44]. Other additional tasks brought about by digitally active patients, such as documenting data in an understandable format for patients [83], communicating asynchronously with patients [44, 84, 85], and acting as a digital tutor [20, 21], may also help explain our results. These new tasks should be recognized in organizations’ work planning. DHTs should only be provided to patients who are suited to digital self-care, and guidelines should be established for organizations to facilitate this assessment [86]. The authorities should increase technical digital support, to which the physician can refer a patient in need of assistance. Moreover, the work and information overload of physicians could be reduced by ensuring that patients use DHTs that are assessed as effective, for which initiatives are underway [87], and by developing methods to filter information that is relevant to patient care from the self-measurement data.

Another interesting result from our study was that the physicians’ experience that the interprofessional collaboration had progressed was related to lower SRIS but greater time pressure. The association with lower SRIS may indicate that some physicians have good user experience of DHTs, meaning they find these technologies easy to use and effective for professional communication and data sharing, previously shown to promote interprofessional collaboration [88–90]. The association with increased time pressure is somewhat surprising, as interprofessional collaboration supported by digitalization has been expected to reduce and allocate tasks [89, 91]. However, the findings of a review [92] suggest that professionals may not yet know how to work effectively in joint decision-making or coordination of care, and learning remote collaboration may be time-consuming. Organizations should therefore plan good practices for interprofessional collaboration [92, 93].

Our study found an association between a considerable amount of digital work and all job strain outcomes. The use of several HISs as a stressor is consistent with previous studies [30, 33, 44–46], and based on our results, continued efforts are needed to promote HISs interoperability. To our knowledge, our results on teleconsultations as a stressor bring new insights to the research field. For example, physicians who frequently conduct teleconsultations can have on average a 47% greater chance of psychological stress compared to those who perform teleconsultations only to some extent. This result conveys previous work-life research that has suggested an association between extensive technology exposure in general and employee stress [13, 47, 48].

Our results may be partly explained by the COVID-19 pandemic, during which teleconsultations were rapidly adopted [50, 52] and not everyone was qualified to conduct them [94]. The digital competence needs for teleconsultations include knowledge and skills about information security and management, strategies for digital communication and patient engagement, and effective utilization of telemedicine technologies, as well as the motivation to encounter patients remotely [94, 95]. After 2020 in Finland, teleconsultation education has been included in the national medical curriculum [96] and training has been organized for practicing physicians [97]. Internationally, there is evidence of the benefits of comprehensive teleconsultation training on physicians’ stress [39]. Employers should require their staff to participate in these training courses during working hours, as voluntary courses might not be conducive to busy work schedules or reach individuals with a critical perception of digitalization [98]. The effects on the well-being at work should also be monitored.

Our study suggested that among physicians who frequently conduct teleconsultations, the greatest time pressure may be experienced by those with fewer than 6 years of work experience. The result corroborates previous studies of early-career physicians, in which time management challenges have also been found to be common in traditional consultations [99, 100] and documentation work [101, 102], and preparedness for teleconsultations has been perceived as weak [103]. Thus, although early-career physicians tend to have grown up in the information age, they should not be expected to adapt easily to teleconsultations. Inexperience may complicate the assessment of a patient’s condition via a remote connection and patient description within the limited time available. Therefore, it is essential that employers support the development of teleconsultation capabilities by organizing effective time management training comprising time management tools, practical advice, and real-life applications, for example [99].

### Limitations

This study has some limitations. The small R^2^ values (8–41%) of the models suggest that the independent variables could not comprehensively explain the variance in physicians’ job strain. Although we adjusted the analysis for several factors, it is likely that adding potential confounders from factors that were not captured by the survey, such as leadership and those related to the personal lives of the physicians, would have rendered the analysis more reliable. It should be noted that the respondents could have interpreted the response option ‘disagree’ differently in positively worded statements about work change. However, disagreement with several statements was associated with a greater job strain compared to a neutral perception. As a result, we interpreted that the ‘disagree’ response meant a change to work in the opposite direction to the presented statement. Moreover, momentary stressors at work may have possibly affected the responses. We were still able to adjust the analysis for the limited experience in the use of the current EHR system, which can be stressful [30, 74]. Causality cannot be established in our cross-sectional data; therefore, future research may employ longitudinal design, utilizing organizational absenteeism records and well-being surveys.

The 25% response rate to our survey is a limitation, but the rate is similarly low in previous studies targeting physicians [104]. The low response rate may, in part, be attributed to the common challenge related to surveys distributed via email, where not all messages may have been successfully delivered, or where email addresses could have changed. Our sample included the experiences of almost every fourth Finnish physician and is estimated to be representative, although older physicians, specialists, and those working in hospitals may have been slightly overrepresented [57, 105]. The generalizability of our results might be less robust when considering younger physicians, non-specialists, and those working in public health centers in Finland. Caution must be taken with regard to the generalizability of the results to other countries with different health systems. As digitalization has accelerated worldwide [50], the means to prevent possible adverse effects on physicians’ well-being at work should also be considered in other countries.

## Conclusions

Physicians may experience job strain when the amount of digital work is significant, and the changes in work do not align with the goals of healthcare digitalization or streamline tasks. The goals of future digitalization should better consider the physician’s routine work, and not only focus on the health system perspective. Physicians’ perceptions of goals not being realized and greater job strain may be related to the use of HISs that disrupt clinical encounters, reminders embedded in HISs that do not support decision-making, and insufficient competence in teleconsultations. Nevertheless, our results suggest that a portion of physicians was satisfied with the changes due to digitalization, which may have alleviated their job strain. This emphasizes how important it is to foster physicians’ positive perceptions of digitalization through training and improving DHTs. Teleconsultation training focusing on time management could promote the well-being of early-career physicians in particular. Patients adopting a more active role and changes in interprofessional collaboration with digitalization may have increased physicians’ workloads, which should be recognized in work planning. We recommend investing in physicians’ well-being in digital work to ensure the success of digitalization and the retention of competent and committed physicians.

### Supplementary Information

**Additional file 1.** Study variables.

**Additional file 2.** Missing data and multiple imputation.

## Data Availability

The datasets used and analyzed during the current study are available from the corresponding author on reasonable request, subject to approval by the study group and the Finnish Medical Association.

## Abbreviations

ANOVA: Analysis of Variance
AI: Artificial Intelligence
CDSS: Clinical Decision Support System
CI: Confidence Interval
DHT: Digital Health Technology
EHR: Electronic Health Record
HIS: Health Information System
OR: Odds Ratio
SRIS: Stress Related to Information Systems

## References

[1] Prasad K, Poplau S, Brown R (2020). Time pressure during primary care office visits: a prospective evaluation of data from the healthy work place study. J Gen Intern Med.

[2] Rotenstein LS, Torre M, Ramos MA (2018). Prevalence of Burnout among physicians: a systematic review. JAMA.

[3] Shanafelt TD, Boone S, Tan L (2012). Burnout and satisfaction with work-life balance among US physicians relative to the general US population. Arch Intern Med.

[4] Cooper CL, Dewe PJ, O’Driscoll MP (2001). Organizational stress: A review and critique of theory, research, and applications.

[5] Arnsten AFT, Shanafelt T (2021). Physician distress and burnout, the neurobiological perspective. Mayo Clin Proc.

[6] Dewa CS, Loong D, Bonato S (2017). The relationship between physician burnout and quality of healthcare in terms of safety and acceptability: a systematic review. BMJ Open.

[7] Tsiga E, Panagopoulou E, Sevdalis N (2013). The influence of time pressure on adherence to guidelines in primary care: an experimental study. BMJ Open.

[8] Williams ES, Rathert C, Buttigieg SC (2020). The personal and professional consequences of physician burnout: a systematic review of the literature. Med Care Res Rev MCRR.

[9] European Commission, Directorate-General for Employment, Social Affairs and Inclusion, McGrath J. Analysis of shortage and surplus occupations 2020. Publications Office of the European Union; 2020. 10.2767/933528

[10] Eurofound, Wilczynska A, Cabrita J, Parent-Thirion A. Sixth European working conditions survey – Overview report (2017 update). Publications Office of the European Union; 2017. 10.2806/422172

[11] Karasek RA (1979). Job demands, job decision latitude, and mental strain: implications for job redesign. Adm Sci Q.

[12] Demerouti E, Bakker AB, Nachreiner F, Schaufeli WB (2001). The job demands-resources model of burnout. J Appl Psychol.

[13] Eurofound. Telework and ICT-based mobile work: Flexible working in the digital age. Publications Office of the European Union; 2020. 10.2806/337167

[14] Fernemark H, Skagerström J, Seing I, Ericsson C, Nilsen P (2020). Digital consultations in Swedish primary health care: a qualitative study of physicians’ job control, demand and support. BMC Fam Pract.

[15] European Commission, Directorate-General for Health and Food Safety. Opinion on assessing the impact of digital transformation of health services. Publications Office of the European Union; 2019. 10.2875/09099

[16] World Health Organization. Global strategy on digital health 2020–2025. World Health Organization; 2021. https://apps.who.int/iris/handle/10665/344249. Accessed 9 Nov 2022.

[17] Meskó B, Drobni Z, Bényei É, Gergely B, Győrffy Z (2017). Digital health is a cultural transformation of traditional healthcare. mHealth.

[18] World Health Organization. Classification of digital health interventions. World Health Organization; 2018. http://apps.who.int/iris/bitstream/handle/10665/260480/WHO-RHR-18.06-eng.pdf?sequence=1. Accessed 4 Apr 2022.

[19] Ministry of Social Affairs and Health. Information to support well-being and service renewal. eHealth and eSocial Strategy 2020. Ministry of Social Affairs and Health; 2015. http://urn.fi/URN:ISBN:978-952-00-3575-4

[20] Vallo Hult H, Hansson A, Svensson L, Gellerstedt M (2019). Flipped healthcare for better or worse. Health Inform J.

[21] Kaihlanen A-M, Laukka E, Nadav J, Närvänen J, Saukkonen P, Koivisto J (2023). The effects of digitalisation on health and social care work: a qualitative descriptive study of the perceptions of professionals and managers. BMC Health Serv Res.

[22] Sinsky C, Colligan L, Li L, Prgomet M, Reynolds S, Goeders L (2016). Allocation of physician time in ambulatory practice: a time and motion study in 4 specialties. Ann Intern Med.

[23] Mann DM, Chen J, Chunara R, Testa PA, Nov O (2020). COVID-19 transforms health care through telemedicine: evidence from the field. J Am Med Inform Assoc JAMIA.

[24] Sutton RT, Pincock D, Baumgart DC, Sadowski DC, Fedorak RN, Kroeker KI (2020). An overview of clinical decision support systems: benefits, risks, and strategies for success. Npj Digit Med.

[25] Day A, Scott N, Kevin Kelloway E (2010). Information and communication technology: Implications for job stress and employee well-being. In New Dev Theor Concept Appr Job Stress.

[26] Saukkonen P, Elovainio M, Virtanen L, Kaihlanen A-M, Nadav J, Lääveri T (2022). The interplay of work, digital health usage, and the perceived effects of digitalization on physicians’ work: network analysis approach. J Med Internet Res.

[27] McBride S, Alexander GL, Baernholdt M, Vugrin M, Epstein B (2023). Scoping review: Positive and negative impact of technology on clinicians. Nurs Outlook.

[28] Heponiemi T, Hyppönen H, Kujala S, Aalto A-M, Vehko T, Vänskä J (2018). Predictors of physicians’ stress related to information systems: a nine-year follow-up survey study. BMC Health Serv Res.

[29] Heponiemi T, Hyppönen H, Vehko T, Kujala S, Aalto A-M, Vänskä J (2017). Finnish physicians’ stress related to information systems keeps increasing: a longitudinal three-wave survey study. BMC Med Inform Decis Mak.

[30] Heponiemi T, Kujala S, Vainiomäki S, Vehko T, Lääveri T, Vänskä J (2019). Usability factors associated with physicians’ distress and information system-related stress: cross-sectional survey. JMIR Med Inform.

[31] Golz C, Peter KA, Zwakhalen SMG, Hahn S (2021). Technostress among health professionals - a multilevel model and group comparisons between settings and professions. Inform Health Soc Care.

[32] Melnick ER, Dyrbye LN, Sinsky CA, Trockel M, West CP, Nedelec L (2020). The association between perceived electronic health record usability and professional burnout among US physicians. Mayo Clin Proc.

[33] Vainiomäki S, Aalto A-M, Lääveri T, Sinervo T, Elovainio M, Mäntyselkä P (2017). Better usability and technical stability could lead to better work-related well-being among physicians. Appl Clin Inform.

[34] Weigl M, Beck J, Wehler M, Schneider A (2017). Workflow interruptions and stress atwork: a mixed-methods study among physicians and nurses of a multidisciplinary emergency department. BMJ Open.

[35] Crampton NH, Reis S, Shachak A (2016). Computers in the clinical encounter: a scoping review and thematic analysis. J Am Med Inform Assoc JAMIA.

[36] Kilponen K, Huhtala M, Kinnunen U, Mauno S, Feldt T (2021). Illegitimate tasks in health care: Illegitimate task types and associations with occupational well-being. J Clin Nurs.

[37] Moy AJ, Hobensack M, Marshall K, Vawdrey DK, Kim EY, Cato KD (2023). Understanding the perceived role of electronic health records and workflow fragmentation on clinician documentation burden in emergency departments. J Am Med Inform Assoc JAMIA.

[38] Poly TN, Islam MM, Yang HC, Chuan Y, Li J (2020). Appropriateness of overridden alerts in computerized physician order entry: systematic review. JMIR Med Inform.

[39] Alkureishi MA, Choo Z-Y, Lenti G, Castaneda J, Zhu M, Nunes K (2021). Clinician perspectives on telemedicine: observational cross-sectional study. JMIR Hum Factors.

[40] Gomez T, Anaya YB, Shih KJ, Tarn DM (2021). A qualitative study of primary care physicians’ experiences with telemedicine during COVID-19. J Am Board Fam Med.

[41] Hilty DM, Crawford A, Teshima J, Chan S, Sunderji N, Yellowlees PM (2015). A framework for telepsychiatric training and e-health: Competency-based education, evaluation and implications. Int Rev Psychiatry.

[42] Haverfield MC, Tierney A, Schwartz R, Bass MB, Brown-Johnson C, Zionts DL (2020). Can patient-provider interpersonal interventions achieve the quadruple aim of healthcare? A systematic review. J Gen Intern Med.

[43] Worley L, Stonnington C, LoboPrabhu S, Summers RF, Moffic HS (2019). A model for maintaining well-being and preventing burnout for psychiatrists. Combating Physician Burnout: A Guide for Psychiatrists.

[44] Eriksson P, Hammar T, Lagrosen S, Nilsson E (2022). Digital consultation in primary healthcare: the effects on access, efficiency and patient safety based on provider experience; a qualitative study. Scand J Prim Health Care.

[45] Vainiomäki S, Heponiemi T, Vänskä J, Hyppönen H (2020). Tailoring EHRs for specific working environments improves work well-being of physicians. Int J Environ Res Public Health.

[46] Vehko T, Hyppönen H, Ryhänen M, Tuukkanen J, Ketola E, Heponiemi T (2018). Tietojärjestelmät ja työhyvinvointi – terveydenhuollon ammattilaisten näkemyksiä [Information systems and wellbeing at work – views of health care professionals. Finn J EHealth EWelfare.

[47] Babbott S, Manwell LB, Brown R, Montague E, Williams E, Schwartz M (2014). Electronic medical records and physician stress in primary care: results from the MEMO Study. J Am Med Inform Assoc JAMIA.

[48] Berg-Beckhoff G, Nielsen G, Ladekjær LE (2017). Use of information communication technology and stress, burnout, and mental health in older, middle-aged, and younger workers – results from a systematic review. Int J Occup Environ Health.

[49] Brady D, Kuiper E. Addressing the challenges of the healthcare workforce: Ensuring the future of health in Europe. Brussels: European Policy Centre; 2023. https://www.epc.eu/content/PDF/2023/CHES_PB.pdf. Accessed 6 Oct 2023.

[50] Keesara S, Jonas A, Schulman K (2020). Covid-19 and health care’s digital revolution. N Engl J Med.

[51] European Commission. Digital Economy and Society Index (DESI) 2021: Finland. European Commission; 2021. https://ec.europa.eu/newsroom/dae/redirection/document/88700. Accessed 8 Aug 2022.

[52] Reponen J, Keränen N, Ruotanen R, Tuovinen T, Haverinen J, Kangas M. Tieto- ja viestintäteknologian käyttö terveydenhuollossa vuonna 2020: Tilanne ja kehityksen suunta [Use of information and communications technology in Finnish health care in 2020. Current situation and trends]. Finnish Institute for Health and Welfare (THL); 2021. https://urn.fi/URN:ISBN:978-952-343-771-5

[53] Haverinen J, Keränen N, Tuovinen T, Ruotanen R, Reponen J (2022). National development and regional differences in ehealth maturity in Finnish public health care: survey study. JMIR Med Inform.

[54] Kenkimäki H, Keränen N, Haverinen J, Reponen J (2021). EHR-connected specialty specific auxiliary systems in public specialized healthcare 2014–2020. Finn J EHealth EWelfare.

[55] Duodecim. Duodecim’s system that warns physicians about potential mistakes receives notable recognition in Estonia. 2021. https://www.duodecim.fi/english/2021/01/29/duodecims-system-that-warns-physicians-about-potential-mistakes-receives-notable-recognition-in-estonia/. Accessed 10 Sep 2022.

[56] Sotkanet. Vacant physicians’ positions at municipal health centres (shortage of doctors) as a percentage of the total number of positions for physicians at municipal health centres, %. Finnish Institute for Health and Welfare (THL); 2023. https://sotkanet.fi/sotkanet/en/index? (Shortage of doctors, Hospital district).

[57] Finnish Medical Association. Physicians 2019. Finnish Medical Association; 2019. https://www.laakariliitto.fi/site/assets/files/5256/sll_taskutilasto_en_220620.pdf. Accessed 6 Mar 2022.

[58] Keskimäki I, Tynkkynen L-K, Reissell E, Koivusalo M, Syrjä V, Vuorenkoski L, et al. Finland: Health System Review 2019. 2019;21:196. https://iris.who.int/handle/10665/327538. Accessed 6 Mar 2022. 31596240

[59] Finnish Institute for Health and Welfare. Information management in social welfare and health care. 2022. https://thl.fi/en/web/information-management-in-social-welfare-and-health-care. Accessed 21 Aug 2022.

[60] Vehko T. E-health and e-welfare of Finland: Check Point 2022. Finnish Institute for Health and Welfare (THL); 2022. https://urn.fi/URN:ISBN:978-952-343-891-0

[61] Finnish Medical Association. Electronic health record systems as a tool for physicians 2021 (Questionnaire). 2021. https://www.laakariliitto.fi/site/assets/files/5229/electronic_health_record_systems_as_tool_for_physicians_2021_questionnaire.pdf. Accessed 27 Aug 2022.

[62] Viitanen J, Hyppönen H, Lääveri T, Vänskä J, Reponen J, Winblad I (2011). National questionnaire study on clinical ICT systems proofs: physicians suffer from poor usability. Int J Med Inf.

[63] Hyppönen H, Kaipio J, Heponiemi T, Lääveri T, Aalto A-M, Vänskä J (2019). Developing the national usability-focused health information system scale for physicians: validation study. J Med Internet Res.

[64] Finnish National Board on Research Integrity. The ethical principles of research with human participants and ethical review in the human sciences in Finland: Finnish National Board on Research Integrity TENK guidelines 2019. Finnish National Board on Research Integrity; 2019. https://tenk.fi/sites/default/files/2021-01/Ethical_review_in_human_sciences_2020.pdf. Accessed 12 Apr 2022.

[65] Harris PE (1989). The nurse stress index. Work Stress.

[66] Kivimäki M, Lindström K (1992). Työstressi ja hyvinvointi hoitoalalla: kyselylomakkeiden kehittely [Job stress and well-being of care providers: development of a standardized survey instrument]. Hoitotiede.

[67] Elo A-L, Leppänen A, Jahkola A (2003). Validity of a single-item measure of stress symptoms. Scand J Work Environ Health.

[68] Financial Supervisory Authority. Työttömyyskassat: Tilastot kassoittain 2019 (excel) [Unemployment funds: Statistics by funds 2019 (excel)]; 2020. https://www.finanssivalvonta.fi/tilastot/vakuutus/tyottomyysvakuutus/. Accessed 26 Feb 2022.

[69] IBM Corporation. Manual: IBM SPSS Missing Values 28. 2021. https://www.ibm.com/docs/en/SSLVMB_28.0.0/pdf/IBM_SPSS_Missing_Values.pdf. Accessed 26 Feb 2022.

[70] van Ginkel JR, Linting M, Rippe RCA, van der Voort A (2020). Rebutting existing misconceptions about multiple imputation as a method for handling missing data. J Pers Assess.

[71] Rubin DB (1987). Multiple Imputation for Nonresponse in Surveys.

[72] Harrell FE, Harrell FE (2015). Multivariable Modeling Strategies. Regression Modeling Strategies: With Applications to Linear Models, Logistic and Ordinal Regression, and Survival Analysis.

[73] Hoff T, Lee DR (2021). Burnout and physician gender: what do we know?. Med Care.

[74] Heponiemi T, Gluschkoff K, Vehko T, Kaihlanen A-M, Saranto K, Nissinen S (2021). Electronic health record implementations and insufficient training endanger nurses’ well-being: cross-sectional survey study. J Med Internet Res.

[75] Maldonado G, Greenland S (1993). Simulation study of confounder-selection strategies. Am J Epidemiol.

[76] Kirkwood BR, Sterne JAC (2003). Essential Medical Statistics.

[77] Virtanen L, Kaihlanen A-M, Laukka E, Gluschkoff K, Heponiemi T (2021). Behavior change techniques to promote healthcare professionals’ eHealth competency: a systematic review of interventions. Int J Med Inf.

[78] Stadin M, Nordin M, Broström A, Magnusson Hanson LL, Westerlund H, Fransson EI (2016). Information and communication technology demands at work: the association with job strain, effort-reward imbalance and self-rated health in different socio-economic strata. Int Arch Occup Environ Health.

[79] Ragu-Nathan TS, Tarafdar M, Ragu-Nathan BS, Tu Q (2008). The consequences of technostress for end users in organizations: conceptual development and empirical validation. Inf Syst Res.

[80] Embi PJ, Leonard AC (2012). Evaluating alert fatigue over time to EHR-based clinical trial alerts: findings from a randomized controlled study. J Am Med Inform Assoc JAMIA.

[81] Bu F, Fancourt D (2021). How is patient activation related to healthcare service utilisation? Evidence from electronic patient records in England. BMC Health Serv Res.

[82] Greene J, Hibbard JH, Sacks R, Overton V, Parrotta CD (2015). When patient activation levels change, health outcomes and costs change, too. Health Aff (Millwood).

[83] Delbanco T, Walker J, Bell SK, Darer JD, Elmore JG, Farag N (2012). Inviting patients to read their doctors’ notes: a quasi-experimental study and a look ahead. Ann Intern Med.

[84] Fiske A, Buyx A, Prainsack B (2020). The double-edged sword of digital self-care: Physician perspectives from Northern Germany. Soc Sci Med.

[85] Glock H, Milos Nymberg V, Borgström Bolmsjö B, Holm J, Calling S, Wolff M (2021). Attitudes, barriers, and concerns regarding telemedicine among Swedish primary care physicians: a qualitative study. Int J Gen Med.

[86] Silven AV, van Peet PG, Boers SN, Tabak M, de Groot A, Hendriks D (2022). Clarifying responsibility: professional digital health in the doctor-patient relationship, recommendations for physicians based on a multi-stakeholder dialogue in the Netherlands. BMC Health Serv Res.

[87] Haverinen J, Turpeinen M, Falkenbach P, Reponen J (2022). Implementation of a new Digi-HTA process for digital health technologies in Finland. Int J Technol Assess Health Care.

[88] Hujala A, Taskinen H, Oksman E, Kuronen R, Karttunen A, Lammintakanen J. Sote-ammattilaisten monialainen yhteistyö: Paljon palveluja tarvitsevat asiakkaat etusijalle [A multiprofessional collaboration of health and social care professionals: Prioritising customers with high need of services]. Yhteiskuntapolitiikka. 2019;84:5–6. https://urn.fi/URN:NBN:fi-fe2019112744444

[89] Barr N, Vania D, Randall G, Mulvale G (2017). Impact of information and communication technology on interprofessional collaboration for chronic disease management: a systematic review. J Health Serv Res Policy.

[90] Rawlinson C, Carron T, Cohidon C, Arditi C, Hong QN, Pluye P (2021). An overview of reviews on interprofessional collaboration in primary care: barriers and facilitators. Int J Integr Care.

[91] Reeves S, Freeth D, Leathard A (2003). New forms of technology, new forms of collaboration?. Interprofessional Collaboration.

[92] van Huizen LS, Dijkstra PU, van der Werf S, Ahaus K, Roodenburg JL (2021). Benefits and drawbacks of videoconferencing for collaborating multidisciplinary teams in regional oncology networks: a scoping review. BMJ Open.

[93] Soukup T, Lamb BW, Arora S, Darzi A, Sevdalis N, Green JS (2018). Successful strategies in implementing a multidisciplinary team working in the care of patients with cancer: an overview and synthesis of the available literature. J Multidiscip Healthc.

[94] Sharma R, Nachum S, Davidson KW, Nochomovitz M (2019). It’s not just FaceTime: core competencies for the medical virtualist. Int J Emerg Med.

[95] Jidkov L, Alexander M, Bark P, Williams JG, Kay J, Taylor P (2019). Health informatics competencies in postgraduate medical education and training in the UK: a mixed methods study. BMJ Open.

[96] Veikkolainen P, Tuovinen T, Jarva E, Tuomikoski A-M, Männistö M, Pääkkönen J (2023). eHealth competence building for future doctors and nurses – Attitudes and capabilities. Int J Med Inf.

[97] University of Oulu. Etälääkäritoiminnan koulutus [Telemedicine training]. 2021. https://www.oulu.fi/fi/yliopisto/tiedekunnat-ja-yksikot/laaketieteellinen-tiedekunta/etalaakaritoiminnan-koulutus. Accessed 2 Mar 2023.

[98] Foadi N, Varghese J (2022). Digital competence - a key competence for todays and future physicians. J Eur CME.

[99] Pitre C, Pettit K, Ladd L, Chisholm C, Welch JL (2018). Physician Time Management. MedEdPORTAL J Teach Learn Resour.

[100] Miles S, Kellett J, Leinster SJ (2017). Medical graduates’ preparedness to practice: a comparison of undergraduate medical school training. BMC Med Educ.

[101] Holmgren AJ, Lindeman B, Ford EW (2021). Resident physician experience and duration of electronic health record use. Appl Clin Inform.

[102] Wu A, Parris RS, Scarella TM, Tibbles CD, Torous J, Hill KP (2022). What gets resident physicians stressed and how would they prefer to be supported? A best–worst scaling study. Postgrad Med J.

[103] Wong CJ, Nath JB, Pincavage AT, Bird A, Oyler JL, Gill K (2022). Telehealth attitudes, training, and preparedness among first-year internal medicine residents in the COVID-19 era. Telemed E-Health.

[104] Cunningham CT, Quan H, Hemmelgarn B, Noseworthy T, Beck CA, Dixon E (2015). Exploring physician specialist response rates to web-based surveys. BMC Med Res Methodol.

[105] Finnish Institute for Health and Welfare. Electronic health records as professionals’ tools. 2022. https://sampo.thl.fi/pivot/prod/en/steps2/laakari/summary_tiiviste6. Accessed 21 Aug 2022.

