# Supplementary material for: Associations of perceived changes in work due to digitalization and the amount of digital work with job strain among physicians: a national representative sample
Source: BMC Med Inform Decis Mak. 2023 Nov 8;23:252. doi: 10.1186/s12911-023-02351-9 (PMC10631156; doi:10.1186/s12911-023-02351-9)
Supplement: Supplementary file 1 — Additional file 1. Study variables. [file 12911_2023_2351_MOESM1_ESM.docx]

# Additional file 1: Study variables

Study variables were derived from an online survey [1] conducted as part of the *Electronic Health Record Systems as a Tool for Physicians 2021 Study* [2].

## Dependent variables

*Stress related to information systems (SRIS)*

In this survey we wish to find out any connection between health information systems and your wellbeing at work. How often has each of the issues mentioned below clearly disturbed, worried, or burdened you at work during the past 6 months?

1. Changing health information systems
2. Difficult, poorly functioning information technical equipment/software

Response options:

1. Very rarely or never
2. Quite rarely
3. Every once in a while
4. Quite often
5. Very often or constantly

*Time pressure*

In this survey we wish to find out any connection between health information systems and your wellbeing at work. How often has each of the issues mentioned below clearly disturbed, worried, or burdened you at work during the past 6 months?

1. Constant rush and pressure due to uncompleted work
2. Not enough time to perform work properly

Response options:

1. Very rarely or never
2. Quite rarely
3. Every once in a while
4. Quite often
5. Very often or constantly

*Psychological stress*

Stress means a situation where a person feels tense, restless, nervous, or anxious or finds it hard to sleep because they constantly worry about things. Do you feel such stress now?

Response options:

1. Not at all
2. Just a little
3. To some extent
4. Quite a lot
5. Very much

Recorded as 0=no (response options 1–2) and 1=yes (3–5).

## Independent variables

*Perceived changes in work due to digitalization (6 variables)*

Electronic healthcare services have increased. EHR systems have been used for a long time but, in addition, patients are increasingly often offered electronic services, such as self-care services, teleconsultation, appointment scheduling, and services for recording and viewing their data.

How has such digitalization of healthcare affected your work? Please assess the change during the past three years.

1. Patients have assumed a more active role in their treatment.
2. It has become easier to obtain information on patients.
3. Intelligent decision support systems support a physician’s work.
4. Consultations with patients have become faster.
5. Interprofessional collaboration has progressed.
6. Possibilities for preventive work have improved.

Response options:

1. Fully agree
2. Somewhat agree
3. Neither agree nor disagree
4. Somewhat disagree
5. Fully disagree

Recoded as 1=neutral (response option 3), 2=agree (1–2), and 3=disagree (4–5).

*Number of HISs in daily use*

How many different clinical systems do you log into daily in your clinical work?

Response options:

1. 0
2. 1
3. 2
4. 3
5. 4
6. 5 or more

Recoded as 1=Two systems or fewer and 2=Three or more.

*Frequency of teleconsultations*

Does your main employment involve teleconsultation with patients (remote treatment by phone, chats, video contact, other electronic contact)?

Response options:

1. Not at all
2. A little
3. To some extent
4. Much
5. Very much

Recoded as 1=not at all–to some extent and 2=much–very much.

## Background variables

*Age*

Year of birth (please select).

Encoded as 1=younger than 35 years, 2=35–44 years, 3=45–54 years, 4=55–64 years. Age was only used for descriptive purposes due to its correlation with the length of work experience.

*Gender*

Response options:

1. Male
2. Female
3. Other

*Length of work experience*

Basic medical education: year of completion (please select).

Encoded as 1=2–5 years, 2=6–9 years, 3=10–19 years, and 4=20 years or longer.

*Experience with the current EHR*

How long you have used the EHR system you MAINLY use in your employment?

Response options:

1. More than 6 years
2. 4–6 years
3. 1–3 years
4. 6 months–less than a year

Recoded as 1=longer than 3 years, 2=1–3 years, and 3=less than 1 year. The responses could indicate how long time ago the EHRs had been implemented at the respondents’ workplace or the respondents had started to work at their current workplace.

EHR grade

On a scale of 4 to 10 (with 4 being the lowest score and 10 being the highest score) how would you rate the EHR you mainly use?

Recoded as 1=low grade (grade 4–7), 2=high grade (8–10), and 2=no opinion.

*Working unit*

When doing clinical work do you MAINLY work:

Response options:

1. On an inpatient ward?
2. In outpatient care, in an outpatient clinic or doctor’s practice?
3. In an emergency department?
4. In an emergency medical service?
5. In administration?
6. In diagnostic medical specialties – e.g. radiology, laboratory medicine, pathology?
7. In the operating theatre?
8. In an intensive care or monitoring room?
9. In the delivery room?

Recoded as 1=inpatient, 2=outpatient, 3=emergency department, 4=operative, intensive care, or delivery room, 5= diagnostics (i.e., radiology, laboratory medicine, or pathology), and 6=administrative.

*Working sector*

Place of main employment.

Response options:

1. University central hospital
2. Central hospital
3. Other public hospital (regional hospital, municipal hospital)
4. Municipal health center, welfare center or station
5. Private medical station, center, or hospital
6. Government
7. Other

Recoded as 1=public hospital, 2=public health center, 3=private clinic or hospital, and 4=other, which included student health services and government.

*Location of employment*

Location of your main employment, hospital district (please select).

Based on the six catchment areas for tertiary care in Finland. The respondents from Åland were recoded with the nearest area of the mainland, Turku University Hospital.

## Additional file 1 References

1 Finnish Medical Association. Electronic health record systems as a tool for physicians 2021 (Questionnaire). 2021. https://www.laakariliitto.fi/site/assets/files/5229/electronic_health_record_systems_as_tool_for_physicians_2021_questionnaire.pdf (accessed 27 Aug 2022).

2 Finnish Institute for Health and Welfare. Information management in social welfare and health care. 2022. https://thl.fi/en/web/information-management-in-social-welfare-and-health-care (accessed 21 Aug 2022).
