# Supplementary material for: Associations of perceived changes in work due to digitalization and the amount of digital work with job strain among physicians: a national representative sample
Source: BMC Med Inform Decis Mak. 2023 Nov 8;23:252. doi: 10.1186/s12911-023-02351-9 (PMC10631156; doi:10.1186/s12911-023-02351-9)
Supplement: Supplementary file 2 — Additional file 2. Missing data and multiple imputation. [file 12911_2023_2351_MOESM2_ESM.docx]

# Additional file 2: Missing data and multiple imputation

Study variables were derived from an online survey [1] conducted as part of the *Electronic Health Record Systems as a Tool for Physicians 2021 Study* [2].

Of the studied physicians, 8% (*n* = 324/4271) had incomplete data with at least one value missing. The proportion of missing values were highest (≥ 2%) for supported decision-making (*n* = 156), improved possibilities for preventive work (*n* = 112) and accelerated clinical encounters (*n* = 87). Independent-samples *t* tests and χ2 tests of independence showed that there were significant differences between the complete and incomplete cases in SRIS, time pressure, length of work experience, gender, and working unit. Thus, a missing at random phenomenon was assumed [3].

We employed multiple imputations (*n* = 5 datasets) using an automatic method [4] for all study variables to generate valid statistical inferences for missing data [5]. Analysis with multiple imputations did not result in substantial differences compared to complete case analysis, partly due to the relatively small proportion of missing data. Thus, all significant results in the pooled analysis were also significant in the original data and vice versa, including similar effect sizes. However, the multiple imputations helped to standardize the sample size and simplify the interpretation of results. The method also prevented dropping those respondents out of the final analysis who lacked a response for only a few variables, so the valuable views of each respondent could be considered. All the displayed data in the results section was derived from the means of the estimates in imputed datasets to obtain a pooled estimate [6].

## Additional file 2 References

1 Finnish Medical Association. Electronic health record systems as a tool for physicians 2021 (Questionnaire). 2021. https://www.laakariliitto.fi/site/assets/files/5229/electronic_health_record_systems_as_tool_for_physicians_2021_questionnaire.pdf (accessed 27 Aug 2022).

2 Finnish Institute for Health and Welfare. Information management in social welfare and health care. 2022. https://thl.fi/en/web/information-management-in-social-welfare-and-health-care (accessed 21 Aug 2022).

3 Little RJA, Rubin, DB. *Statistical Analysis with Missing Data*. Hoboken, NJ: John Wiley & Sons 2002.

4 IBM Corporation. Manual: IBM SPSS Missing Values 28. 2021. https://www.ibm.com/docs/en/SSLVMB_28.0.0/pdf/IBM_SPSS_Missing_Values.pdf

5 van Ginkel JR, Linting M, Rippe RCA, et al. Rebutting Existing Misconceptions About Multiple Imputation as a Method for Handling Missing Data. *J Pers Assess* 2020;102:297–308. doi:10.1080/00223891.2018.1530680

6 Rubin DB. *Multiple Imputation for Nonresponse in Surveys*. Hoboken, NJ: John Wiley & Sons 1987.
